# 

**Published:** 1900-10-06

**Authors:** 


### The Hospital

A JOURNAL OF

THE MEDICAL SCIENCES AND HOSPITAL ADMINISTRATION,

INCLUDING

A SPECIAL SECTION FOR NURSES.

IN TWO VOLUMES ANNUALLY.

15 a

EDITED BY

SIR HENRY BURDETT, K.C.B.,

AND

SOLOMON C. SMITH, M.D.

LIBRARY

SURGEON GENERAL'S OFFICE

OCT.-29. 1901

174878.

VOLUME XXIX.

October, 1900, to March, 1901.

London:

PUBLISHED BY THE SCIENTIFIC PRESS (LIMITED), AT THE HOSPITAL BUILDING,
28 & 29 SOUTHAMPTON STREET, STRAND, W.C.

MDCCCCI.

WI
H069X

### INDEX TO VOL. XXIX., 1901.

*** Articles under the general heading *Hospital Clinics and Medical Progress* are distinguished by the initial (C.) for *Hospital Clinics*, and by (P.) for the rest of the series. (W.) distinguishes the *Institutional Workshop* articles, and (A.) the *Annotations*.

Abdominal Incisions, Closing of (P.), 17
Abdominal Operations. *See p.* 293
Abscess after Appendicitis (C.), 418
Abscess, Sub-diaphragmatic (C.), 30
Acland, The late Sir Henry (A.), 43
Actinomycosis (C.), 65, 261
Actions at Law, 313
Adiposis dolorosa (P.), 263
Adulteration of Food, 378
Albuminuria, Cystic (P.), 280
Alcoholic Cirrhosis of the Liver in an Infant (P.), 82
Amenorrhœa, Primary, Prognosis in (C.), 349
American "Political Hospitals" (A.), 61
Anæmia, Pernicious (P.), 335, 335
Anæmia, Splenic (P.), 385
Anæsthesia, Accidents during (P.), 33
Anæsthesia, Local (P.), 33
Anæsthetics, The Administration of (P.), 119
Anæsthetics Committee, Report of the (A.), 314
Anæsthetics, Effects of (P.) 249
Anæsthetics, Progress in (P.), 33
Aneurysm (P.), 67, 351
Aneurysm, Thoracic, Treatment of (C.), 171
Angina Pectoris (P.), 324
Anopheles, The resting Position of (C.), 63
Antiseptics (P.), 173
Antitoxin Treatment of Diphtheria, The (C.) 343
Appendicitis (P.) 49, (C.), 98, (P.), 193, 316
Argyll-Robertson Pupil, The (P.), 263
Army Medical Corps, The Future of the 377
Arsenic—Was it Arsenic after all? (A.), 327
Arsenic in Beer (C.), 365
Arsenic in Food and Drink, 273
Arsenic in Medicine (A.), 184
Arsenic plus Alcohol as a Cause of Neuritis (C.), 277
Arsenical Beer, The Sale of (C), 314
Arsenical Poisoning in Beer Drinkers (P.), 437
Ascites, Chylous (P.), 212
Asthma (P.), 281
Asthma, Treatment of (P), 281
Asylum Matters (W.), 144, 162
Ataxia, Permanent Non progressive (P.), 263
Athens, The Hospitals of (W.), 36
Bacillus Ærogenes Capsulatus (P.), 84
Bacillus Coli (P.), 404
Bacillus Typhosus and Allied Organisms (P.), 83
Bacillus, Tubercle (P.), 4[illegible]3
Bacteria in Human Milk (P.), 83
Bacteria, Separation of, from Milk (P.), 8[illegible]
Bacteriology, Progress in (P.), 83, 264, 403
Beer Question, The, 202
Biopsies (A.), 379
Birmingham Consultative Institute, The (A.), 415
Birmingham Medical and Surgical Consultative Institution (A.), 95
Bishop's Pub, The (A.), 95
Bladder, Exstrophy of the (P.), 266
Bladder, Tuberculous Disease of the (P.), 266
Blood Pressure (P.), 51
Blood, Progress in Diseases of the (P.), 334, 385
Books, Notes on, 122
Bookworld (*Books, Magazines, and Pamphlets Reviewed*)
Baruch, Principles and Practice of Hydrotherapy, 86
Blackadder. *See Savile*
British Sanatoria Annual, 1901, 250
Burdett-Coutts, The Sick and Wounded in South Africa, 267
Butlin, Diseases of the Tongue, 19
Carless. *See Rose*
Carwardine, Operative and Practical Surgery, 53
Celli (trans. by Eyre), Malaria according to new Researches, 141
Cheadle, On some Cirrhoses of the Liver (Lumleian Lectures, 1900) 3[illegible]1
Christy, Mosquitos and Malaria, 441
Conjugal Intercourse and Chastity (leaflets), 177
Cook. Children of Scorn and Medical Papers, 177
Cowen. Electricity in Gynæcology, 53
Curtis. Essentials of Practical Bacteriology, 231
Densmore. Consumption and Chronic Disease, 159
Eade. The Norfolk and Norwich Hospital, 1770 to 1900, 159
Eccles. Hernia, 19
Ewart. First Stage Botany, 122
Eyre. *See Celli*
Fox. Photographic Atlas of the Disease of the Skin, 441
Gould. Student's Medical Dictionary, 141
Haldane. How we Escaped from Pretoria, 177
Book World (*continued*)—
Horne. The Mirage of two Buried Cities, 250
Hutchinson. Food and the Principles of Dietetics, 267
James, Emily (edit. by). Englishwoman's Year Book, 1901, 250
Jefferson. China and the Present Crisis, 195
Jellett. A Short Practice of Gynæcology, 122
Kelynack and Kirby. Arsenical Poisoning in Beer Drinkers, 301
Macalister. Memoir of James Macartney, 388
Mackay. History of Ancient Gynæcology, 319
Manson. Tropical Diseases, 70
Metropolitan Hospitals and Vivisection, 388
Murrell. What to do in Cases of Poisoning, 319
Parkes. Hygiene and Public Health, 354
Payne. Thomas Sydenham, 104
Rose and Carless. Manual of Surgery, 70
Rosslyn (Earl of). Twice Captured, 122
Savile and Blackadder. A Prescriber's Companion, 319
Spencer. Consumption, 177
Stedman (edit. by) Twentieth Century Practice, 195
Stewart. Manual of Physiololgy, 159
Tuckey. Treatment by Hypnotism and Suggestion, 104
Walsh. Golden Rules of Skin Practice, 231
Who's Who, 1901, 250
Zorn. Bunce, the Bobby and the Broads, 195
Bright's Disease, The Clinical Varieties of (C.), 261
Bristol, New Infirmary at (W.) 427
British Medical Association, A Reformed, 339
Bronchitis (P.), 16, 281, 403
Brow Presentations (P.), 155
Cæsarian Section (P.), 405
Calf Lymph, The Use of, in Epidemic Small-pox (C.), 401
Camp Sanitation (A.), 131
Cancer (P.), 157
Cancer, The Cause of (C) 365
Cancer, Progress in (P.), 120, 137
Cancer of the Uterus, Recent Observations on (C.), 293
Cancerous and other Tumours, The Causation of (C.), 434
Cardiac Disease, The Causes of in Middle and Advanced Life (C.), 419
Catheter Cytoscope, The (P.), 263
Cautery, The Actual (C.), 418
Census, The, and Density of Population (A.), 239
Cerebro-Spinal Meningitis (P.), 193
Cerebro-Spinal Surgery, Progress in (P.), 421
Cheyne-Stokes Respiration (P.), 282
Chinese Labour in England, The Question of (A.), 26
Chloretone (P.), 283
Chloroform Administration, Dosage in (C.), 383
Chlorosis (P.), 386
Chlorosis, The Medicinal Treatment of (C.), 279
Cholæmia (P.), 191
Cholelithiasis (P.), 66
Chorea, A Case of, illustrating its Neuropathic Heredity (C.), 420
Chronic Arsenical Poisoning, 148
Cisterns (A.), 379
Civil Surgeons and the War, 147
Clinical Characters associated with certain Types of Degeneracy (C.), 45
Cocaine Anæsthesia during Labour (C.), 147
Cocaine, Economy in (C.), 189
Coffee Poisoning (C.), 295
Cold Bath as a Means of Diagnosis, The (C.), 153
Colitis (P.), 190
College Woman's Influence on the Race, The (A.), 43
Colon, Hypertrophic Dilatation of the, in Infants (P.), 82
Colonial Troops Entertainment, 179
Communion Cup, The (A.), 185
Competition in Fees (A.), 2
"Consent," A Question of (A.), 77
Constipation (C.), 207
Consumption, A Sanatorium for: Hailey on the Chilterns (W.), 161
Consumptives, State Care of (P.), 228
Creosote in Whooping Cough (C.), 99
Cruro-Scrotal Hernia, A Case of (C.), 399
Cunningham and the *Daily Express*, 274
Curious Vacation Course, A (A.), 2
Curtains and Mosquitoes (A.), 27
Cystitis caused by Bacillus Pyocyaneus (P.), 266
Cystoscopy (P.), 248
Cystostomy, Abdominal (C.), 171
Dangerous Trades, 87, 105, 123, 142, 160, 196
Day Terrors (P.), 139
Dermatology, Periscope of (P.), 367
"Detective" Questions in Life Assurance "Forms" (A.), 345
Diabetes, The Nervous Origin of (C.), 348
Diabetes, Progress in (P.), 296
Diabetic Coma, Progress in (P), 102
Diagnosis, Mistaken (C.), 364
Diagnosis, Science and Instinct in (C.), 313
Digestive Organs, Progress in Disease of the (P.), 157, 172, 190, 212
Diphtheria, The Antitoxin Treatment of (C.), 348
Diphtheritic Heart (P.) 68
Diphtheroid Bacilli in Milk (P.), 83
Diploma in Public Health, The (A.), 167
Disappointments in Surgery (C.), 224
Dog's Ears and Thumb Licking (A.), 27
Dormiol (P.), 283
Drink and the Barmaid (A.), 449
Duodenal Ulcer (P.), 315
Dust and Disease, 123
Dyeing and Dry Cleaning, 193
Dysentery (P.), 212
Dysmenorrhœa (P.), 420
East Sussex, The New Asylum for (W.), 303
Eclampsia, Puerperal (P.), 387
Eczema (P.), 367
Editor's Letter Box, 39, 57, 109, 126, 217, 271, 34[illegible], 3[illegible]5
Electric Trams and County Boundaries (A.), 167
Electricity, the Domestic Uses of (A.), 113
Embolism of the Pulmonary Artery (P.), 282
Emphysema (P.), 137
Empyema following Lobar Pneumonia (C.), 117
Endocarditis (P.), 52
Enteric Fever in Childhood (C.), 115, 133
Enteric Fever in South Africa (C.), 116, 335
Enuresis, The Neurotic Form (P.), 120
Epilepsy, Surgical Treatment of (P.), 422
Epileptics in Colonies, Results of the Treatment of (P.), 283
Ethyl Chloride (P.), 33
Excreta, The Immediate Disinfection of Infected (C), 116
Eyes and Teeth, Pathological Relations of (P.), 69
Fæcal Impaction (C.), 365
Fat Friends, Our (A.), 257
Fevers, The Non-malarial, of the Tropics (C.), 31[illegible]
Fevers, Progress in (P.), 84, 101, 118
Fibroma involving the Tympanum (P.), 230
Filatow's or Koplik's Spots (C.), 12
File Cutting, 87
Filth and Fashion (A.), 76
Food Stuff (P.), 212
Foul Mouth, the Consequences of a (C.), 187
Fractures and Dislocations (P.), 173, 210
Free Trade in Lectures (A.), 26
French Medical Men on Sanatoria (A.), 149
Fresh Air and Influenza (A.), 326
Friendly Societies and the Wage Limit (A.), 448
Gall Tracts, Disease of the (P.), 366
Gangrene of the Lung, Bacteriology of (P.), 83
Gannister Disease, 160
Gas and Oxygen, Use of, in Ophthalmic Operations (P.), 69
Gas Stoves (A), 397
Gastrectomy (P.), 17
Gastric Ulcer (P), 17, 299; (C.), 330, 331
Gastric Ulcer, Perforated, Operation for (C.), 153; (P.), 452
Gastro-Enterostomy (P), 300
Gastrotomy for Hæmatemesis (C), 65
Gastrotopsis (P), 17, 172
General Surgery, Progress in (P), 173, 210
Genito-Urinary Surgery, Progress in (P), 265, 332, 436
German Army Medical Service, The (A.), 431
German Measles (A.), 326
Germs, Vitality of (P), 264
Glances at the Hospitals, 24, 33, 56, 73, 91, 109, 125, 162, 233, 266, 321, 355
Glasgow Outbreak, The (P.), 85
Glasgow Royal Infirmary Competition (W.), 371
Glasgow's Opportunity (A.), 3
Glaucoma, Progress in (C.), 48
Glycerine as an Antiseptic (P.), 261
Gonococci, New Stain for (P.), 84
Gout in the Throat and Nose (C.), 225
Grindstones (Dangerous Trades), 105
Gunshot Wounds of the Abdomen (P.), 18
Gynæcology and Obstetrics, International Congress of (C.), 48
Gynæcology, Progress in (P.), 420
Habitation, The Changing Style of (A.), 396

Habitual Inebriates (A.), 203
Hæmorrhage in Gastric Ulcer, Death from (C.), 419
Hammer Toe (P.), 247
Handwriting and Health (A.), 2
Harveian Oration, The (A.), 61
Health Census, A, 255
Health Resorts, Some Notes on (W.), 285
Health and Revenue, 429
Heart, Dilatation of the (P.), 68
Heart Disease, Complications of (P.), 68
Heart, Diseases of the, Progress in (P.), 34, 51, 67, 333, 351, 368
Heart Reflex (C.), 279
Heart, Rupture of the (P.), 67
Heart Sounds (P.), 51
Heart, Wound of the (C.), 189
Hemiplegia (P.), 263
Hernia (P.), 175, 384
Hernia, Cruro Scrotal (C.), 399
Hernia, Femoral, Radical Cure of (P.), 194
Hernia, Gangrenous Inguinal (P.), 384
Hernia, Obturator, Strangulated (P.), 194
Hernia [Radical Cure] (P.), 385
Hernia, Strangulated with Volvulus (P.), 194
Hernia, Umbilical, Radical Cure of (P.), 194
Hip Disease (P.), 247
Homœopathy and Malaria (A.), 149
Homœopathy and Rational Medicine, 256, 341, 375
Home Treatment of Lunatics, The (A.), 345
Hospital Abuse and its Cure (W.), 124
Hospital, An Ill-fated, 448
Hospital Appliances, Some (W.), 424
Hospital and Asylum Construction (W.), 161
Hospital Economics (W), 197
Hospital Festivals, Meetings, &c. (W.), 55, 91, 106, 145, 181, 193, 321, 340, 372, 391, 406, 425, 443, 455
Hospital Saturday Fund (W.), 106
Hospital Sunday Fund (W.), 339, 355
Hospitals and Lay Managers (A.), 27
Hospitals, Permanent Military (W.), 20, and *see p.* 39
Housing of the Working Classes (P.), 350
How to People Africa (A.), 77
Humanitarian and the Hooligan, The (W.), 179
Huxley Lecture, The, 10
Hyperchlorydria (P.), 172
Hypnotic Suggestion (P.), 283
Hypospadias, Operations for (P.), 403
Hysterectomy for Cancer of the Uterus (C.), 135
Imlach, The Case of Dr., 395
Immunity [Bacteriology] (P.), 264
Imperial Yeomanry Hospital at Deelfontein; Notes on Cases (C.), 14
Importance of Research, The (C.), 11
Inebriates, The Provision for (A.), 275
Infants, The Feeding of, in Health and Disease (C.), 381
Infected Air from Small-Pox Hospitals (W.), 443
Infection by Means of Clothing (A.), 414
Inflammable Paints, 142
Inflammation as a Precursor of Tumour Formation (C.), 226
Influenza in Children (C.), 245
Influenza and Enteric Fever, 129
Influenza and Heart Disease (P.), 67
Influenza, The Nervous Complications of (C.), 13
Influenza, and the Nervous System (P.) 192
Inguinal Region, Anatomy of the (P.), 175
Insane, The Story of the, from Year to Year, 57, 125, 145, 163, 234, 286, 322, 357, 411, 4[illegible]5
Insanity in Lead Workers (P.), 283
Institutional Questions (W.), 199 233, 323, 341
International Tabulation of Causes of Death (A.), 221
Intestines (P.), 173
Intestines, Surgery of the (P.), 315
Intra-Abdominal Omental Torsion (P.), 384
Intra-Nasal Treatment of Diseases of the Ear (P.), 229
Intussusception (P.), 316; (C.), 451
Iodine in Diphtheria (C.), 171
Jaundice in Children (C.), 451
Kidney, Cystic (P.), 248
Kidney Disease, Ocular Changes in (P.), 248
Kidney, Movable and Hepatic Colic (C.), 315
Kidneys, Movable (P.), 248
King, The, and the Hospitals (W.), 337
King's College: Opening of New Laboratories (W.), 89
Koplik's, or Filatow's Spots (C.), 12
Laboratory Milk (P.), 82
Labour, Normal (C.), 99
Lead and Metallic Poisons, The Toxic Action of (P.), 317
League of Mercy, The (W.), 180, 216, 375
Legal Intelligence (W.), 162
Lesson from Denmark, A (A.), 361
Leukæmia (P.) 385
"Life Capital," 238
Light Baths (C.), 262
Liver, Diseases of the (P.), 191
Liver and Gall-Bladder, &c., Surgery of the (P.), 66, 366
Liver, Hydatid Cysts of the (P.), 366
Liver, Neoplasms of the (P.), 66
Liver, Rupture of the (P.) 366
Liverpool Central Home for District Nurses (W.), 390
London Municipalities, The, 59
London School of Tropical Medicine, 447
London, The University of (A.), 397
Lunacy Law (A.), 414
Lungs, Diseases of the (P.), 422, 429, 454
Malaria (P.), 118; (C.), 206, 407
Malaria: A Crucial Experiment (A.), 3
Manchester Health Missionaries 54
Massage, Direct, of the Heart in Chloroform Poisoning (C.), 330
Mastoid Operation, The Radical (P.), 230
Mastoid, Sarcoma of (P.), 230
Measles Complicated with Meningitis and Spinal Myelitis (P.), 140
Measles, Whooping Cough, and Education (A.), 296
Meat Inspection (A.), 113
Meckel's Diverticulum (P.), 316
Medical Army Reserve (A.), 201
Medical Council and the Curriculum, The, 183
Medical Council and Dr. Irvine, The, 165
Medical Education, 111
Medical Ethics (C.), 46
Medical Grievances. *See* p. 126
Medical Knowledge, The Popularisation of, 25
Medical Session, The, 1
Medical Societies now Existing in London, Some Account of the More Important, 5
Medical Student, The Over-examined (A.), 26
Medical Treatment at Isolation Hospitals, 112
Medicine, Progress in (P.), 136, 281
Medullary Narcosis during Labour (P.), 387
Melancholia, The Treatment of (C.), 46
Memory, On Some Aspects of, 268, 284, 302, 320, 370, 389, 440
Mental Disorder and Toxæmia (C.), 152
Mercurol, The Use of, in Urethritis (P.), 352
Methylated Spirit, The Drinking of (A.), 257
Metropolitan Asylums Board of London and its Work (W.), 37
Middle Ear, Chronic Suppuration of the (P.), 229
Midwives Question at Manchester, The (A.), 167
Military Dietetics (C.), 98
"Milk! Below," 413
Modern Sociology, 54, 71, 87, 105, 123, 142, 160, 196, 232
Morphia, Toxic Action of (C.), 31
Motto for the New Year, A, 220
Mouth and Œsophagus (P.), 157
Municipal Bakeries (A.), 369
Municipalisation of Hospitals, The (A.), 275
Musculo-Spiral Paralysis from Fractured Humerus (P.), 422
Myasthenia gravis (P.), 209
Myotonia congenita [Thomsen's Disease] (P.) 140
National Hospital for the Paralysed and Epileptic (W.), 455
Nationalisation of the Children, The, 278
Nerve-Ganglia, Surgery of (P.), 421
Neuritis, Alcoholic, Sir T. Lauder Brunton on (C.), 190
Neuritis, Peripheral, The Epidemic of (C.), 188
Neurology, Progress in (P.), 192, 209, 263, 283, 437
Neuroses of Childhood, The Commoner (P), 121
New Appliances and Things Medical, 18, 35, 85, 103, 121, 140, 158, 176, 194, 249, 283, 300, 353, 369, 405
Notes and News, 4, 28, 44, 62, 78, 96, 114, 132, 150, 168, 186, 204, 222, 240, 258, 276, 292, 310, 328, 346, 362, 380, 398, 416, 432, 450
Nurse who Insists, The (A.), 345
Obstetrics, Progress in (P.), 154, 386, 405
Ocular Affections, Differentiation of (P.), 69
Olive Oil Injection in Typhoid Fever (C.), 119
Oophorectomy for Diseases of the Breast (C.), 66
"Open-Sesame" (A.), 239
Open-air Treatment of Consumptive Paupers (A), 42
Ophthalmology, Progress in (P.), 68, 156
Opium in Cancer of the Uterus (C.), 435
Orthopædics, Progress in (P), 227, 247
Ovariotomy in the Eightieth Year (C.), 436
Ovary, The Internal Secretion of the (C.), 47
Overcrowding and Consumption (C.), 314
Pancreas (P.), 191
Pancreatic Surgery (P.), 366
Paralysis, Infantile (P.), 247
Pay Hospitals in England (W.), 183
Pediatrics, Progress in (P.), 82, 100, 119, 139
People's Restaurants (W.), 269
Pericarditis (P.), 52
Pericarditis, Suppurative (C.), 246
Peritoneum, Progress in Surgery of the (P.), 298
Peritonism (C.), 134
Peritonitis (P.), 298
Peritonitis, Diffuse Septic (P.), 17
Peritonitis, Tuberculous (P.), 18, 100; (C.), 452
Pharmacopœia An Imperial (A.), 203
Phthisis (P.), 208
Phthisis—Is Wetness of Soil a Cause of Phthisis? (C.), 382
Phthisis, Country (C.), 135
Physical Therapeutics, 166
Physician and Surgeon (C.), 118
Placenta prævia (P.), 154
Plague, The (P.), 84, (A.), 397
Plague, Antidotes to (C.), 79
Plague, The Diagnosis of (C.), 29
Plague, The Signs and Symptoms of (A.), 61
Plague, at Hull, The (A.), 291, 345
Pleura, Diseases of the (P.), 136
Pleurisy (P.), 454
Pneumonia (P.), 15, 282, 454
Pneumonia, Lobar, and Enteric Fever (P.), 100
Pneumo-thorax (P.), 137
Polyclinic, The (A.), 431
Polyorromenitis (C.), 226
Post-graduate Study in London (W.), 21
Post-partum Hæmorrhage (P.), 156
Practical Departments, 23
Practitioner, The Duty of the, in Acute Intestinal Obstruction (C.), 134
Præfrontal and Cerebellar Tumours, The Differential Diagnosis of (C.), 347, 363
Precautions against Plague (A.), 431
Pregnancy, Primary Ovarian (C.), 277
Prince, The, and the Hospitals, 219
Prince of Wales's Hospital Fund, The (W.), 145 213
Professional Reticence (A.), 431
Promises to pay (C.), 81
Propriety in Small Things (A.), 351
Prostate, Enlargement of the (P.), 331
Prostatectomy in two Stages (C.), 81
Pyschiatry, Progress in (C.), 31, 51, 317
Ptosis of the Liver (P.), 191
Puerperal Eclampsia (C.), 117
Puerperal Sepsis (P.), 405
Puerperal Septicæmia (P.), 155
Pulmonary Œdema (P.), 137
Pulmonary Tuberculosis, Some Serious Modes of Onset in (C.), 170
Punjab, Dispensaries of the (W.) 233
"Pure and Wholesome Water," A Legal View of (C.), 312
Purification by Fire (C.), 116
Pylorus, Benign Obstruction of the (P.), 17
Pyrexia, The Cause of the, in Fever, 308
Quackery, The Suppression of (A.), 327
Queen, The Death of the, 289
Queen-Empress, The Late, and After, 290
Quinine Pills (C.), 452
Racial and other Immunity to Fever (A.), 360
Radcliffe Infirmary, Oxford, The (W), 356
Rate-supported Institutions (W.), 144
Rating of Hospitals, The (W.), 199
Rats and Plague (A.), 77
Rectum, Prolapse of the (P.), 100, 436
Rectum, Stricture of the (P.), 436
Rectum, Wounds of the (P), 436
Renal Medicine, Progress in (P), 248, 280
Respiratory System, Diseases of the (P.), 15, 208
Returning Troops, The (W.), 108
Revolt of the Colleges, The (A.), 95
Rheumatic Fever (P.), 83, (C.), 259
Rheumatism and Gout (P.) 453
Rhythm and Blood (P.), 51
Ringworm Infection from Animals (C.), 335
Roentgen Society, The (C.), 99
Rosebery's (Lord) Rectorial Address (A.), 131
Round Worms (C.), 64
Rumination in a Boy (P.), 82
Running Water (C.), 207
Sanitary Standard, Wanted a (A.), 203
Sanitation and the Motorists (A.), 239
Scandal of "Hospital Scandals," The (W.), 143
Scarlet Fever (P.), 83, (C.), 205, 223, 244
Scarlet Fever Hospital Isolation in (C.), 382
Scarlet Fever, Return Cases of (C.), 400
Scarlet Fever, Two Attacks of, in the same Patient within a few Months (C.), 399
Scattered Homes for Poor Law Children (A.), 185
School Board Elections, 93
Sclerosis, Insular, in a Child: Clinical Lecture (C.), 433
Sex, The Origin of (C.), 206
Shellfish and Enteric Fever (A.), 339
Sight, The Training of (C.), 99
Skiagraphy in the Diagnosis of Chest Disease (C.), 315
Skiagraphy in the Diagnosis of Urinary Calculi, (C.), 295
Slum Proprietor, The Rights of the (A.), 113
Small-pox, the Germ of, and Vaccinia (C.), 400
Small-pox in Scotland (A.), 415
Smegma Bacillus (P.), 281
Sociology, *See Modern Sociology*
Soda-water Bottles, 232
Something Like a Jockey Club! 185
South African Hospitals Commission, The, 307
South African War Story, A (A.) 379
Spending Department, A (A.), 449
Spinal Curvature (P.), 227
Splenectomy (P.), 366
Sponges, Purification of, by Boiling (C.), 401
Spoon-shaped Indentations in the Skulls of the Newly Born (C.), 295
Sprains, The Immediate Treatment of (C.), 136
Stands Glasgow where it did? (A.), 131
State Medicine, Progress in (P.), 228, 350
Sterilisation of Rubber Gloves, The (C.), 153
Sterilisation of Water, The Extemporaneous (A.), 309
Stillbirth, The Hydrostatic Test of (C.), 190
Stomach, Dilatation of the (P.), 17, 172, 299
Stomach, Endoscopy of the (P.), 299
Stomach, Surgery of the (P.), 16, 299
Stomach and Œsophagus, Foreign Bodies in the (P.), 299

Story of a Dummy Corpse (A.), 360
Strabismus, The Operative Treatment of (P.), 68
Street Architecture, 130
Streptococcus (P.), 83
Stridor, Congenital (O.), 170
Student of Medicine, The, 24, 40, 58, 73, 92, 110, 128, 164, 182, 200, 217, 236, 272, 288, 306, 324, 342, 358, 393, 412, 446, 464
Sub-diaphragmatic Abscess (P.), 299
Surgery, Cerebro-spinal (P.), 421
Surgery, General (P.), 173, 210
Surgery, Genito-urinary and Rectal (P.), 265, 331, 352, 436
Surgery of the Intestines (P.), 315
Surgery of the Liver, &c. (P.), 66, 366
Surgery of the Peritoneum (P.), 17, 208
Surgery, Progress of (P.), 194, 229, 315, 384, 402, 436
Surgery, Rectal (P.), 402
Surgery of the Stomach (P.), 16, 299
Surgery of the Ureters (P.), 265
Surgery of the Vermiform Appendix (P.), 49, 316
Syphilis, Tertiary, in the Nose and Pharynx (O.), 314
Syphilitic Joint Disease (O.), 401
Teaching of Surgery in America, The (O.), 244
Teeth of the Nation, The (A.), 257
Temperance Reform in Kansas (A.), 415
Testicle, Misplaced and Undescended (P.), 402
Testicle, Tumours of the (P.), 402
Testicles and Vesiculæ Seminales, Tuberculosis of (P.), 352
Tetanus (P.), 404
Tetanus in Diphtheria Antitoxin (O.), 412
Therapeutic Notes, 48, 246
Therapeutics (P.), 282, 368
Toxæmia and Insanity, 237
Toxic Action of Cocaine and other Drugs (P.), 50
Toxins, The Selective Action of, on the Nervous Tissues,
Tracing the Doctor (A.), 76
Training of the Officer, The (A.), 149
Trial of New Forms of Treatment, The (W.), 285
Tubercle Bacillus, A Direct Attack on the (C.), 226
Tubercle and Smegma Bacilli (P.), 264
Tuberculosis, The Hydriatic Treatment of (C.), 188
Tuberculosis in Prisons (A.), 184
Tuberculous Meat (A.), 221
Tumours complicating Pregnancy (P.), 386
Typhoid (P.), 101
Typhoid and the Tea-kettle (A.), 76
Typhoid Fever in South Africa (O.), 433
Typhoid Fever, The Treatment of (O.), 329
Typhoid Ulcer, Operation for (O.), 262
Ulcer of the Duodenum (P.), 173, 315
Ulcer of the Stomach (P.), 172
Ulnar Nerve, Recurrent Luxation of the (P.), 422
Uræmia (P.), 280
Ureters, Surgery of the (P.), 265
Urethra, Stricture of the (P.), 332
Urinary Pigments (P.), 280
Urology (P.), 249
Urotropine: a Urinary Antiseptic (O.), 294
Uterus, Chronic Cervical Catarrh of the (O.), 63, 79, 97, 151, 169
Uterus, Malignant Disease of the (P.), 420
Uterus, Retroflexion of the Gravid (O.), 207
Uterus, Rupture of the (P.), 154
Vaccine Lymph, Bacillus of (P.), 265
Vermiform Appendix, Surgery of the (P.), 49, 316
Victoria, The Charitable Institutions of (W.), 56
Victoria, Factory Legislation in, 71
Vomiting (P.), 18
"Wart" Story, Another (O.), 13
Water Filtration in America (W.), 221; (O.), 262
Water London and the Housing Question (A.), 430
Whooping Cough (P.), 265
Williams (Sir John) at Cardiff, 42
Wind Exposure and Phthisis, 60
Women Workers, 75
Workhouse Diets (W.), 72
Wounds of the Heart (C.), 245
X Rays and Light, Treatment by (P.), 367
Year and the Century, The, 141
Yellow Fever (O.), 89; (A.), 275; (O.), 435
Yellow Fever Question, The (O.), 117

### THE SPECIAL SECTION FOR NURSES.

Across the Seas, 10
Age 18 81, 241
Alexandra Nurses, The, 211
Amateur Nurse at the Front, An, 170
Appointments, 21, 32, 47, 57, 69, 80, 91, 107, 120, 131, 140, 160, 176, 190, 215, 223, 238, 250, 265, 287, 303, 315, 325, 340
Appointments, Minor, 21, 32, 45, 57, 69, 80, 91, 119, 131, 160, 176, 188, 199, 215, 227, 239, 250, 263, 272, 285, 299, 310
Artistic Novelties, 175
Autumn Novelties for Nurses, 20
Beef-tea for the Sick, Preparations of, 171, 213
Bit of Black Ribbon, A, 229
Bloemfontein to Pretoria in a Hospital Train, From, 53
Book and Its Story, A, 35, 82, 148, 173, 231, 279, 301, 338
Bristol General Hospital, Prize Distribution at the, 160
Burdett-Coutts, M.P., Mr., and "THE HOSPITAL" *Nursing Mirror*, 199
Chelsea Infirmary, Resignation of the Matron of, 216
Christmas Books, 141, 158
Christmas Day, 1900, 140
Christmas Distribution, Our, 156, 176, 191
Christmas in the Hospitals, 183
Christmas Talk with a Patient, A, 169
City Orthopædic Hospital, Lectures to Nurses at the, 254, 289
Creighton, Mrs., on District Nursing, 162
Croydon, An Object Lesson from, 103
Croydon Infirmary, Crisis at, 47, 118, 142, 229
Croydon Infirmary, Crisis at, The Local Government Board and the, 91
Darenth, A Visit to the Schools at, 79
Death in our Ranks, 18, 56, 188, 266, 273, 299, 320
District Nursing Superintendents in Conference, 287
District Nursing, A Personal Experience in, 311
East Preston Guardians and the Superintendent Nurse, The, 250
Echoes from the Outside World, 16, 33, 46, 58, 68, 95, 106, 117, 129, 146, 161, 172, 189, 201, 214, 228, 240, 252, 264, 277, 283, 300, 313, 326, 337
Eden (Lady), on Nursing and Nurses, 156
Everybody's Opinion, 17, 34, 47, 57, 71, 83, 96, 108, 119, 132, 147, 163, 174, 191, 203, 216, 229, 242, 254, 265, 278, 289, 302, 314, 327, 339
Examination Questions for Nurses, 20, 69, 127, 190, 253, 299
First Nurses at Waterval Onder, Transvaal, The, 197
Functions and Entertainments, 130
Funeral Services for Queen Victoria, The, 298
Gastro-Enteritis caused by Sulphuric Fumes, 32
Great Northern Central Hospital, The, 266
Guy's Hospital, 203
Haggerston and Hoxton Nursing Home, Enlargement of, 66
Head Sisters, Lectures to, 335
Heaven-Born Matron, The, 7
Help the Nurses to Help the Sick, 144
Hints to Nurses, 56
THE HOSPITAL Convalescent Fund, 104, 115, 160, 177, 190, 284, 315
Hostel of St. Luke, The, 303
How they Received the Sad News at Vancouver, 262
How to Make a Travelling Tea-Basket, 133
Hypodermic Injections, The Ordering of, 200
Imperial Yeomanry Branch Hospital at Maitland, 84
Important to all Trained Nurses, 28
Isleworth Infirmary, 18
Lectures on Medicine to Nurses, 4, 64, 88, 114, 138, 168
Lectures to Midwifery Nurses, 197, 223, 249
Lectures to Nurses on Medicine and Medical Training, 182, 208, 236, 260, 284, 308, 332
Lectures on Nursing for Probationers, 26, 52, 76, 102, 126, 155
Leicester Infirmary, The Nurses of, 333
Life on a Navvy Settlement, 10
London Temperance Hospital, The Nurses of the, 155
Mafeking Sisters of Mercy, The, 96
Maratha Plague Hospital, Bombay, Sketches from the, 9, 30, 42
Marriage of a Hospital Matron, 32
Medical Lectures to Nurses, 272, 296, 320
Metropolitan Nursing Association, The, 109
My First Week in a London Hospital, 286
National Union of Women Workers, The, at Brighton, 70
Neurotic Diathesis, The, 14
Night in one of Her Majesty's Prisons, A, 13
Nightingale Nurses, The, 18
Not Divided, 176
Notes on News from the Nursing World, 1, 23, 37, 49, 61, 73, 85, 99, 111, 123, 135, 151, 165, 179, 193, 205, 220, 233, 257, 269, 281, 293, 305, 317, 329
Novelties for Nurses, 189, 255
No. 3 Model School Hospital, Pretoria, 211
Nurse in Hot Climates, The, 8, 29, 43, 55, 67, 78, 94, 105, 116, 128
Nurse without a Petticoat, The, 227
Nurses' Bookshelf, The, 107, 144, 188, 212, 241, 276, 334
Nurses' Co-operation, The, 310
"Nurses in Council" and Industrial Law, 56
Nurses and the Industrial Laws, 274
Nurses and Orderlies in South Africa, 64
Nurses, Visiting, for the Middle Classes, 6
Nursing on the *Aurania*, 241
Nursing in British Central Africa, 209
Nursing the C.I.V.'s, 80
Nursing Europeans at Kota Kota, British Central Africa, 309
Nursing in an Indiana Hospital, 145
Nursing Miners in Johannesburg and Soldiers at Sea, 285
Nursing on the *Nubia* Hospital Ship, 103
Nursing in North Germany, 5
Nursing at the Palace of Justice Hospital, Pretoria 77
Nursing at Pinetown Bridge, Natal, 41
Nursing the Plague at Glasgow, 5
Nursing at the Royal Edinburgh Hospital for Sick Children, 44
Nursing at the Temporary International Hospital, Peking, 224
Nursing at Winburg, Orange River Colony, 27
Nursing in Workhouse Infirmaries, 92
Nursing at Wynberg Base Hospital, 139
Nursing, Parish, in Germany, 273
Nursing, Private, in Durban and a Voyage on the *Canada*, 297
Nursing, Private, in Rome, 31
Nursing, Private, in South Africa, 157
Nursing Vocation, The, 335
Ophthalmic Nursing, 225, 239
Patients on the *Persia*, 12
Practical Memorial, A, 273
Presentations, 45, 54, 96, 107, 115, 131, 140, 191, 215, 225, 241, 266, 285, 303, 310, 341
Precepts for "Pros," 298
Queen Alexandra and the Nursing Vocation, 246
Queen Victoria and the Nursing Vocation, 219
Queen Victoria's Jubilee Institute for Nurses, 183
Queen's Commemoration Fund, 312
Resignation of the Lady-Superintendent of Melbourne Hospital, 28
Resignations, 334
Royal British Nurses' Association, 66, 226, 238
Royal British Nurses' Association, New Offices of the, 188
Royal Free Hospital, Nurses' New Quarters at The, 297
Royal National Pension Fund for Nurses, 198, 321
Royal National Pension Fund Nurse's Version of the National Anthem, 250
"St. Andrew's House," The Opening of, 54
St. Mary's Hospital, The Nurses of, 89
Scene on the Solent, The, 251
Scott-Holland, Canon, on Nursing, 263
Scottish Red Cross Hospital, The, 90
Sick Soldiers in South Africa, 237
"Special Nursing," A Word for, 11
Stepney Workhouse, Alleged Neglect at, 327
Travel Notes, 19, 60, 122, 150, 178, 218, 244, 268, 292, 342
Two Hospital Entertainments, 215
Two Pictures from Pretoria, 115
Typical Patients, 15
Uniform on Board Ship, 83
"Visitors for the Unvisited," 237
Volunteer Nursing, A Record of, 275
Wants and Workers, 107, 131, 230, 260, 315, 336
Weddings in the Nursing World, 276
Where to Go, 80, 107, 115, 199, 250, 296, 320, 336
With Lord Roberts from the Cape, 198
Work and Diet of Nurses and Probationers in Hospitals, The, 202
Workhouse Infirmaries, Nursing at the, 261
Working Boys, 176
York County Hospital, 216

Printed by SPOTTISWOODE & CO. LTD., New Street Square, E.C.; and Published by the Proprietors, THE SCIENTIFIC PRESS, LIMITED, at 28 & 29 Southampton Street, Strand, London, W.C.

### NOTICE TO CORRESPONDENTS.

All MSS., Letters, Books for Review, and other matters intended for the Editor should be addressed The Editor, "The Hospital," The Hospital Building, 28 & 29 Southampton Street, Strand, W.C.

The Editor cannot undertake to return rejected MS., even when accompanied by stamped directed envelope.

**Notice to Advertisers and Subscribers.**

All Advertisements, Orders for Copies of The Hospital, and Business Communications should be addressed to **THE MANAGER (not to the Editor)**, The Hospital, The Hospital Building, 28 & 29 Southampton Street, Strand, London, W.C.

The Cost of Subscription, post free, is as follows:—Per week, 2½d.; per per quarter, 2s. 8d.; per half-year, 5s. 3d.; per annum, 10s. 6d. Foreign.—Per annum, 15s. 2d., payable, in all cases, in advance.

**NOTICE.—Vol. XXVIII. of "The Hospital" (April to September, 1900), in handsome cloth binding, will shortly be on sale at the Office of this Journal, price, 7s. 6d. Cases for binding the Numbers contained in this Volume 1s. 6d. Index to Vol. XXVIII., 2 d., post free.**

**The Monthly Part for October is now ready. Price 6d., by post 8d.**

### NOTES AND NEWS.

### GLANCES AT THE HOSPITALS.

### The Student of Medicine.

### APPOINTMENTS.

E. W. Adams, M.B., appointed Medical Officer for the South-East District of the Sheffield Union. A. N. Clemenger, L.S.A., appointed Medical Officer and Public Vaccinator for the Slaidburn District of the Clitheroe Union. C. R. H. Crawford, M.R.C.S., L.R.C.P.Lond., appointed Assistant Medical Officer to the Tonbridge Union Workhouse. F. Richardson Cross, M.B., F.R.C.S., appointed Honorary and Consulting Ophthalmic Surgeon to the Bristol Royal Infirmary. H. Duckworth, M.B., C.M., appointed Medical Officer of Health for the Oswaldwhistle Urban District. R. J. Isaac, L.R.C.P., L.R.C.S.Edin., appointed Medical Officer for the Northern District of the Gower Union. J. C. M. Kinnear, M.B.Aberd., appointed Medical Officer for the Wickham District of the Fareham Union. F. R Mallett, M.D.Edin., L.R.C.P., M.R.C.S., appointed Medical Officer for the Eastern Bolton District of the Bolton Union. Edward J. Moore, M.B., B Ch.Oxf., appointed Medical Officer of Health. H. Gilbert Nicholson, M.R.C.S., L S.A., appointed Surgeon for the Bethnal Green District of the City of London Lying-in Hospital. Alexander Ogilvy, B.S., M.D.,Dub. F.R C.S.I., appointed Honorary Ophthalmic Surgeon to the Bristol Royal Infirmary. J. H. Saunders, M.B., M.R.C.S., appointed Medical Officer in Charge of the Belle-Vue Camp, Simons Town. Nigel Frampton Stallard, M.B., M.R.C.S., L.R.C.P., appointed House Surgeon and Registrar to the Royal Orthopædic Hospital. Courtenay Charles Weeks, M.R.C.S., L.R C.P.Lond., appointed Certifying Factory Surgeon for the Metropolitan Borough of Lewisham.

